# Can public data openness enhance public health resource allocation efficiency? Evidence from the launch of Chinese government data platforms

**DOI:** 10.3389/fpubh.2026.1843304

**Published:** 2026-06-03

**Authors:** Zhenyu Li, Zhao Zhang, Yuning Chu

**Affiliations:** 1 School of Economics, Qingdao University, Qingdao, China; 2 School of Economics and Finance, Xi'an Jiaotong University, Xi'an, China; 3 Department of Clinical Nutrition, the Affiliated Hospital of Qingdao University, Qingdao, China

**Keywords:** data governance, public data openness, public health resource allocation efficiency, quasi-natural experiment, staggered DID

## Abstract

**Introduction:**

Public health resource allocation efficiency constitutes a critical pillar for protecting public health and social welfare. Nevertheless, data silos and information asymmetry constrain the optimization of public health resource allocation.

**Methods:**

Taking the launch of Chinese government data platforms as a quasi-natural experiment, this study uses provincial panel data from 2009 to 2022, adopts DEA, staggered DID and other approaches to investigate the impact and transmission mechanisms of public data openness on public health resource allocation efficiency.

**Results:**

The findings indicate that, first, public data openness has a significantly positive effect on enhancing public health resource allocation efficiency. Second, the policy effect has obvious regional heterogeneity, with a stronger impact on underdeveloped regions. Third, exploratory channel analysis provides suggestive evidence that reducing information acquisition costs and strengthening external oversight may be the critical transmission pathways.

**Discussion:**

Based on these findings, efforts should focus on broadening the scope and deepening the application of data openness. This entails striking a balance between unlocking data value and protecting privacy to ensure coordinated progress. Simultaneously, differentiated regionally strategies should be adopted to promote fair distribution of policy dividends, ultimately enhancing the overall efficiency of public health resource allocation.

## 1 Introduction

Public health resource allocation efficiency is not only a cornerstone for a realizing universal health coverage and promoting Sustainable Development Goal 3 (SDG3) of the UN, but also as a crucial criterion for assessing the effectiveness of public health governance. At present, unbalanced resource allocation and inadequate efficiency have become a common governance challenge that crosses national boundaries (1). Whether the misallocation of medical resources and inadequate primary care services in high-income countries, or the shortage of public health infrastructure and delayed emergency resource dispatching in low- and middle-income economies, the crux of the issue lies in the data silos across departments and sectors - key information such as population health, geographical location, fiscal investment, and emergency reserves are fragmented, preventing policymakers from matching resource supply with the real needs of the public based on accurate data.

With the deep application of digital technology, data have become another vital production factor following land, labor, capital, and technology. Public data, a crucial component of data resources, encompasses not only government administrative data but also data generated by various micro-entities in social activities such as economy, education, and healthcare that are related to public life and interests (2). Owing to its large volume and official attributes, public data plays a dominant role in economic and social datasets (3). As a globally recognized governance innovation, public data openness has been endorsed by international organizations including the UN, OECD, and World Bank as a core approach to break information isolation and unlock data value. To date, over 150 countries and regions worldwide have launched national-level data platforms. Taking China as an example, public data openness has risen to a core reform task at the national strategic level, with the central government issuing numerous supportive policies (4). Local governments in China have successively launched public data platforms since 2012, aggregating massive amounts of data from government and social entities. These platforms play a fundamental role in empowering the real economy, transforming production modes, and enhancing the governance capacity of public sectors, thereby promoting the optimization of public resource allocation from experience-driven to data-driven practices.

From a theoretical perspective, public data openness can provide new technological and institutional support for optimizing public health resource allocation by mitigating information asymmetry, strengthening cross-entity collaboration, and supporting data-driven decision making. However, existing studies have primarily focused on the value of data openness in economic development. To date, no research has systematically clarified the intrinsic relationship between public data openness and public health resource allocation efficiency. There is also a lack of rigorous empirical testing to reveal the mechanism and practical significance of the two.

Based on the above analysis, this study treats the launch of Chinese government data platforms as a quasi-natural experiment, employs provincial-level panel data from 2009 to 2022, applies DEA, staggered DID, and other methods to examine the effects and transmission pathways of public data openness on public health resource allocation efficiency, so as to provide theoretical insights and empirical evidence for countries worldwide, particularly developing economies, to address public health resource allocation dilemmas through data governance and promote the optimization of public health resource allocation.

## 2 An overview of public data openness in China

### 2.1 The realistic background of public data openness in China

Public data openness originates from the international Open Data Movement. Its core principle is for governments to make non-sensitive, reusable public data available to society free of charge or at low cost for applications such as R&D, public services, and commercial applications (5). This has become a globally recognized approach to enhancing government transparency and unlocking the value of data.

In recent years, the Chinese government has attached great importance to public data sharing and openness. Since the 18th National Congress of the Communist Party of China, the relevant policy framework has been continuously improved and refined. Policy documents have established the fundamental principles and implementation pathways for data openness, such as the ““Data Element × ” Three-Year Action Plan (2024–2026)” and the “Regulations on Public Data Sharing and Openness”. In the field of health, supporting instruments such as the “Administrative Measures for Health Statistics” have been introduced to enhance the compliance and orderly nature of public data openness. In terms of policy implementation, since Shanghai and Beijing launched their government data platforms in 2012, local governments at various levels have accelerated the development of public data platforms, continuously increasing the provision of diverse data resources to the public free of charge. According to *the China Local Public Data Openness Utilization Report* (6), by the end of 2024, 243 local governments had launched data platforms, including 24 at the provincial level and 219 at the municipal level. The cumulative volume of data calls exceeded 540 billion, and 111 local governments established data groups to carry out specialized authorized operations on relevant public data.

From a segmented viewpoint, healthcare data plays a core role in public data openness and falls into the category of a “high-value domain”. The current open content mainly covers six major categories: public health data, health resources data, health status data, medical application data, medical payment data, and research innovation data. Typical key terms include the quantity of infectious disease cases, the allocation of medical facilities, outcomes of population health check-up, health surveys of specific groups, usage of healthcare funds, and disease warning. These data are accessible in line with a classified and hierarchical openness principle: macroscopic statistical data are entirely accessible, whereas sensitive data such as individual medical records are supplied in a controlled manner after de-identification, with updates carried out semi-annually or annually.

### 2.2 Literature review

Existing research has mainly concentrated on the economic consequences of public data openness, with a general consensus that such openness exerts positive effects on macroeconomic expansion (7), coordinated regional development (8), and the enhancement of governmental governance efficiency (9). Several studies have explored the value creation mechanism of data by combining endogenous growth theory and human capital theory, indicating that data elements promote economic performance via multiple channels, including advancing the upgrading of industrial structures (10), boosting total factor productivity (11), reducing information processing costs and mitigating uncertainty (12), as well as shaping household consumption and investment choices (13). From a microcosmic viewpoint focusing on enterprises, other studies hold that public data openness helps upgrade corporate production and innovation patterns (14), thereby strengthening firms' green innovation capacity and narrowing the technological disparities between different enterprises (15).

Regarding research on the public health resource allocation efficiency, existing studies have primarily focused on its characteristics of change, spatial disparities, and influencing factors. Research related to China indicates that the growth in output efficiency has slowed in recent years (16). Although disparities in resource allocation efficiency are showing a trend of convergence at the provincial level (17), significant spatial heterogeneity remains (18). Furthermore, some studies have identified issues such as a misalignment between public health resource allocation and regional economic development requirements as well as individual health needs (19, 20). Concerning the influencing factors, relevant studies have mainly approached the issue from the perspectives of internal management and external environment, suggesting that factors such as economic and social development (21), public financial investment (22), and healthcare system reform (23) are significantly correlated with allocation efficiency. Some studies have also focused on the impact of digital technology applications, such as big data and cloud computing, on health resource allocation, attempting to analyze resource allocation optimization from the perspectives of the interactive relationship, internal mechanism, and implementation path between the two (24, 25). However, these studies are mostly normative analysis and case studies, without quantitative findings.

While previous research offers valuable insights for this study, further development can be pursued in the following two areas. First, discussions on the value of public data openness have overly emphasized economic benefits, with few studies paying attention to its social benefits in public services such as healthcare and education. Public data originate from the operational practices of public services and inherently possess the characteristics of public goods. In the healthcare domain, for instance, health departments and medical institutions collect data on patient treatment, health maintenance, and input-output of medical resources, aiming to optimize decision-making and improving service quality. Therefore, analyzing the social benefits of public data openness constitutes an important aspect of realizing its existential value. Second, against the backdrop of digital technology enabling high-quality development in the healthcare industry, studies exploring the relationship between digital resources and health resource allocation are mostly qualitative analyses from the perspectives of institutional frameworks and implementation pathways, with few quantitative investigations addressing this issue.

### 2.3 Contributions

This study takes the launch of Chinese government data platforms as a quasi-natural experiment, focusing on the relationship between public data openness and public health resource allocation efficiency, and systematically elucidates the mechanisms and practical value of the former on the latter. The marginal contributions are primarily reflected in the following aspects. First, building on existing research, this study extends the discussion of public data openness social value to the domain of public health resource allocation efficiency, thereby enriching the analytical perspective of studies on the value-creating role of public data openness. Second, addressing the critical issue of “mismatch between supply and demand” in public health resources, this study constructs a transmission mechanism centered on “information symmetry-collaborative governance-precise allocation”, clarifies the intrinsic logic of data factors empowering public health governance, and offers a Chinese approach for developing countries to enhance the effectiveness of public health governance.

## 3 Research design

### 3.1 Theoretical analysis and research hypothesis

**Figure 1:**
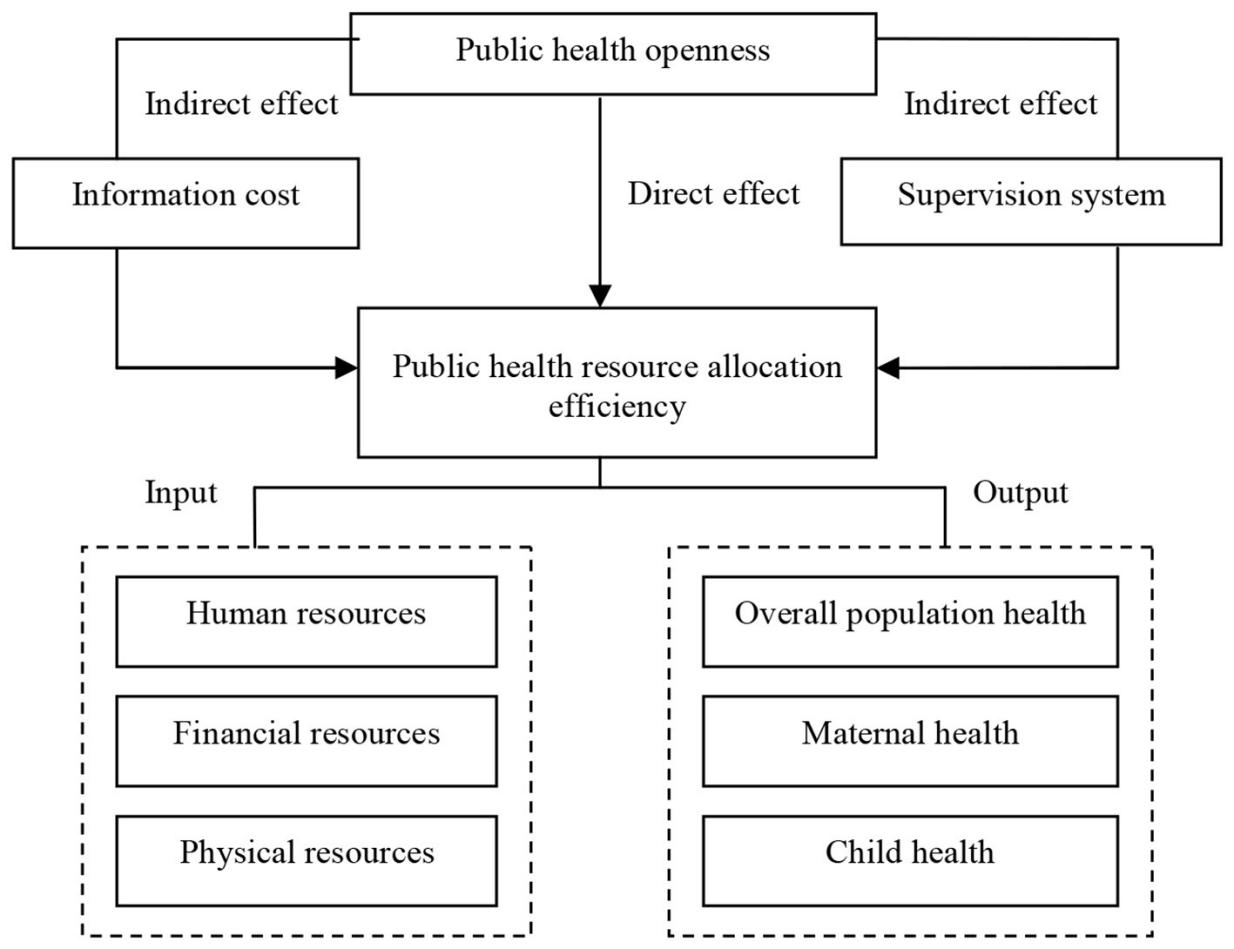
Research roadmap.

Figure 1 is the research roadmap for this study, which mainly involves two issues: first, whether public data openness enhances the public health resource allocation efficiency, second, what the underlying mechanisms are.

First, in terms of direct effects, both factor inputs and outcome outputs in public health resource allocation efficiency rely heavily on multi-dimensional information such as population structure, spatial distribution of diseases, existing health resources, and service utilization. Under the traditional model, data are often fragmented and updated with delays (26). This not only makes it difficult for policymakers to scientifically plan and dynamically adjust health resource distribution, but also prevents medical institutions from accurately matching residents' actual health demands, and ultimately leaves residents with limited access to equitable and accessible public health services. By standardizing, integrating, and openly sharing data on health resources, population health, public services, and geospatial information, public data openness enables real-time visibility and efficient exchange of regional supply and demand information in public health (27). This, in turn, optimizes the spatial allocation and dynamic deployment of core resources such as hospital beds, health workforce, medical equipment, and public health funding, effectively alleviating the structural contradiction between idle resources and supply shortages. Furthermore, data openness can also improve response capacity in public health emergencies, promoting rapid coordination of health resources across regions and departments. Based on this, the following research hypothesis is proposed:

H1: Public data openness can significantly enhance public health resource allocation efficiency.

Second, in terms of transmission mechanisms, public data openness enhances efficiency mainly through two pathways. The first one is to lower the costs of information acquisition. Information asymmetry is a core constraint that hinders public health resource allocation efficiency (28). Data silos force governments, institutions, and social organizations to spend considerable expenses on acquiring effective information (29). For government, the lack of sufficient information makes it hard to accurately identify regional resource gaps and dynamically monitor the status of resource utilization, leading to resource allocation plans that fail to keep pace with actual demands. For residents, limited access to health-related information and the distribution of medical resources may result in irrational healthcare-seeking behaviors, which further exacerbates the mismatch between supply and demand. Reducing information costs transforms the allocation of public health resources from experience-driven to data-driven, thereby effectively alleviating the structural conflict between underutilization of resources and insufficient supply.

The second one involves enhancing oversight mechanisms. According to principal-agent theory, under the traditional model, insufficient transparency regarding key information such as resource input scale, fund utilization details, and service quality outcomes constrains oversight entities from effectively supervising the allocation process (30). Public data openness enhances transparency regarding resource inputs, utilization efficiency, service coverage, and other critical information, thereby establishing a diversified system involving government regulation, social supervision, and public participation. This constrains inefficient resource utilization, reduces rent-seeking and waste, and incentivizes improvements in resource allocation efficiency. Based on this, the following hypotheses are proposed:

H2a: Public data openness can enhance the public health resource allocation efficiency by reducing information acquisition costs.

H2b: Public data openness can enhance the public health resource allocation efficiency by strengthening external oversight mechanisms.

### 3.2 Methods and variable selection

To address the issues proposed in this study, the empirical analysis is structured into three main parts.

First, this study uses DEA to evaluate public health resource allocation efficiency across Chinese provinces. The efficiency score is calculated under a multi-input and multi-output framework, with the detailed indicator system presented in Table 1.

**Table 1:** Indicator system for public health resource allocation efficiency.

Dimension		Indicator	Unit	Direction
Input	Human resource	Number of practicing doctors per 10,000 population	per/10,000	+
		Number of registered nurses per 10,000 population	per/10,000	+
	Financial resource	Per capita government health expenditure	Yuan	+
	Physical resource	Number of primary healthcare institutions per 10,000 population	per/10,000	+
		Number of beds in healthcare institutions per 10,000 population	per/10,000	+
Output	Overall population health	Life expectancy	Age	+
		Mortality rate	‰	-
	Maternal health	Prenatal examination rate	%	+
		Hospital delivery rate	%	+
	Child health	Neonatal visit rate	%	+
		Health care management rate for children under 7 years old	%	+

Public health resource inputs are measured from three dimensions: human, financial, and physical resources. Human resources include the number of practicing physicians and registered nurses per 10,000 population. Financial resources include per capita government health expenditure. Physical resources include the number of primary medical institutions and the number of medical beds per 10,000 population. Focusing on the health improvement orientation of public health resource allocation, this study uniformly defines output indicators as health-related outcomes, covering overall population health, maternal health, and child health. Specifically, overall population health includes life expectancy and mortality rate. Given that mortality rate is a reverse indicator, we adopt a positive transformation of “1- mortality rate” in the empirical analysis. Maternal health includes the prenatal care rate and hospital delivery rate. Child health includes the newborn visit rate and the health care management rate for children under 7 years of age. This indicator system is not only consistent with the intrinsic characteristics of public health resource allocation but also aligns with mainstream practices in the existing literature (31), thus demonstrating sound rationality and feasibility.

Second, given the staggered launch of government data openness platforms across provinces in practice, the launch of such platforms can be considered a policy experiment. Provinces that have launched government data platforms are treated as the treatment group, while those that have not yet launched such platforms serve as the control group. A staggered DID approach is employed to analyze the impact of public data openness on public health resource allocation efficiency.

The model is structured as follows:


$$\begin{array}{l} {EPH}_{it}=\beta_{0}+\beta_{1}DATA_{it}+\beta X+\mu_{i}+\lambda_{t}+\delta_{i}\times t+\varepsilon_{it}\\ \;\left(Model\;1\right) \end{array}$$

In model 1, subscripts *i* and *t* denote province and year. *EPH*_*it*_ represents public health resource allocation efficiency in province *i* during year *t*, which is measured by the efficiency score obtained from the DEA estimation. *DATA*_*it*_ is a proxy for public data openness, which is assigned a value of 1 if province *i* has launched its government data openness platform in year *t*, and 0 if otherwise. *X* denotes a vector of control variables. To isolate the independent effect of public data openness from confounding factors, this study controls for a series of variables reflecting economic development, fiscal conditions and population structure. These variables include per capita GDP (expressed in logarithmic form), local fiscal investment in public health (calculated as the ratio of medical and health expenditure to total general public budget expenditure), fiscal autonomy (calculated as the ratio of general public budget revenue to general public budget expenditure), child dependency ratio, old age dependency ratio, and urbanization rate. We also introduce digital economy policy support intensity as a control variable. Following Jin et al. (32), this variable is measured by the count of digital economy–related keywords, such as digital government, data sharing, smart governance, in annual provincial government work reports. It explicitly captures the time-varying differences in provincial digital governance capacity and policy support intensity, thereby controlling for confounding effects from concurrent digital economy policies. And to a certain extent, it reflects the level of digital development of the province. μ_*i*_and λ_*t*_denote province fixed effects and year fixed effects. To absorb each province's unique linear time-varying path, which effectively mitigates the endogeneity caused by unobserved provincial development trends, we add province-specific time trends δ_*i*_×*t*. ε_*it*_ represents the random error term.

Third, to assess the mediating effect, we employ a two-step approach as outlined by Jiang (33). The model is structured as follows:


$$\begin{array}{l} M_{it}=\beta_{0}+\beta_{1}DATA_{it}+\beta X+\mu_{i}+\lambda_{t}+\delta_{i}\times t+\varepsilon_{it}\;\left(Model\;2\right) \end{array}$$

In model 2, *DATA*_*it*_ remains the proxy for public data openness, which is defined consistently with Model 1. *M*_*it*_ represents the corresponding mechanism variable. If the regression coefficient β_1_ is significant, it suggests the existence of a mediating effect. The definitions of other variables are consistent with those in model 1.

This study divides the impact of public data openness on public health resource allocation efficiency into two main pathways: internal and external. The internal pathway is reflected in the reduction of information costs, whereas the external pathway is reflected in the improvement of supervision system.

While due to data limitations, there are no direct indicators of lower information acquisition costs in the health sector or stronger supervision over health resource allocation. Considering data availability, this study adopts the “Innovation Environment Utility Value” and the “Government Fiscal Transparency Index” as proxy variables to conduct exploratory channel analysis, respectively. The “Innovation Environment Utility Value” is derived from *China Regional Innovation Capability Evaluation Report* published by China Science and Technology Strategy Research Group (34). As a secondary indicator under the comprehensive regional innovation capability index, it is synthesized from multiple metrics reflecting information availability, information processing costs, and technical support, such as the growth rate of telephone subscribers, the number of broadband Internet accesses, highway mileage per 10,000 population, and investment in network infrastructure. It captures regional information infrastructure, communication conditions, and digital access. It can also reflect the overall ease of information acquisition. Thus, it is theoretically relevant to information costs in health resource allocation. Higher value of the indicator indicates lower information acquisition costs. The “Government Fiscal Transparency Index” is derived from *China Fiscal Transparency Report* published by Shanghai University of Finance and Economics Public Policy Research Center (35). This index is constructed based on the disclosure level of government fiscal budgets, public expenditure information, fiscal fund management, and related accountability systems. Fiscal transparency helps strengthen external supervision over the allocation and use of public resources, restrain irregularities in the use of public funds, and thereby theoretically improve the supervision efficiency of health-related fiscal expenditure and resource allocation. Higher values of the indicator indicate stronger constraints on government behavior. Despite the theoretical connection, it should be noted that, as broad provincial-level indexes not specific to the health sector, the two variables cannot directly measure targeted health information disclosure or supervision, which may lead to a relatively weak explanatory power.

### 3.3 Dataset

Given that provincial governments in China had generally launched government data platforms by 2023, effective control groups were no longer available. This study provincial uses panel data covering 29 provinces in Mainland China (excluding Xinjiang and Tibet) from 2009 to 2022. The launch dates of provincial government data platforms were obtained primarily through online searches, with specific launch times as follows: Beijing and Shanghai in 2012; Zhejiang in 2015; Guangdong and Guizhou in 2016; Henan, Jiangxi, Ningxia, Shandong, and Shaanxi in 2018; Fujian, Hainan, Jiangsu, Sichuan, and Tianjin in 2019; Guangxi, Hubei, Hunan, and Qinghai in 2020; Anhui, Gansu, Hebei, and Chongqing in 2021; and Liaoning in 2022. Unless otherwise specified, other data were obtained from *the China Statistical Yearbook, China Fiscal Yearbook*, and *China Health Statistics Yearbook* for the respective years.

## 4 Results

### 4.1 Descriptive analysis

**Figure 2:**
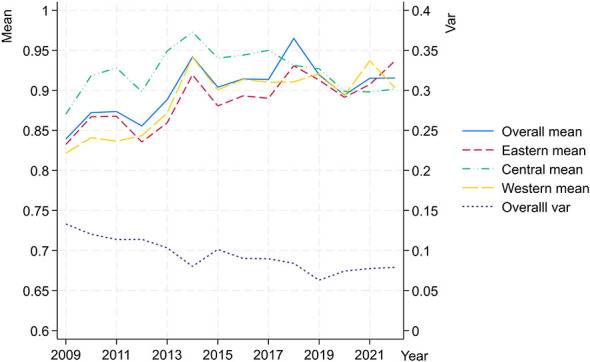
Trend of public health resource allocation efficiency in China.

Table 2 reports the descriptive statistics of the variables, and Figure 2 reports the trend of public health resource allocation efficiency in China, based on the DEA scores. Four key findings can be obtained as follows.

**Table 2:** Descriptive statistics of variables.

Variable	Number	Mean	SD	Min	Max
Public health resource allocation efficiency	406	0.9008	0.0934	0.5852	1
Public data openness	406	0.2709	0.4449	0	1
Per capita GDP (ln)	406	10.7739	0.5253	9.2885	12.1547
Financial investment in public health	406	0.0767	0.0163	0.0397	0.1392
Fiscal autonomy	406	0.4971	0.1900	0.1500	0.9300
Child dependency ratio	406	22.7911	6.2634	9.60	38.30
Old age dependency ratio	406	15.3131	4.2585	7.40	28.80
Urbanization rate	406	0.5868	0.1271	0.30	0.90
Digital economy policy support intensity	406	16.0418	12.0741	0	60
Innovation environment utility value	406	28.1865	9.4876	11.6900	61.7100
Fiscal transparency	406	52.0241	18.5385	10.87	89.38

First, the core characteristic of overall public health resource allocation efficiency is fluctuating upward growth. In 2009, the average efficiency value was approximately 0.84. It climbed steadily and peaked in 2018. Although there is a slight decrease afterwards, the efficiency score remains above 0.90. This means the overall efficiency of public health resource allocation in China has been continuously improving during the study period. Second, from a regional perspective, the efficiency performance of the three major regions exhibits distinct differences and a converging trend. The central region has consistently led in efficiency, with its peak efficiency reaching nearly 0.97 around 2014. The trend in the eastern region closely mirrors the national average. In 2009, the western region had the lowest initial efficiency score among the three, yet it has shown an impressive momentum of catching up, and its efficiency level has been progressively approaching those of the eastern and central regions. Third, regarding inter-provincial differences, the SD has been continuously decreasing from 2009 to 2019. This shows that the divergence in interprovincial allocation efficiency diminished steadily. The distribution of resources became more balanced. Although the variance rebounded slightly after 2019, it was still much lower than the level of 2009, while the overall trend of equalization remained unchanged. Fourth, according to the descriptive statistics of other variables, the SD of each variable is smaller than its mean value. The pronounced heterogeneity in variables such as public data openness and innovation environment may provide important clues for explaining regional efficiency differentials and their dynamic changes.

### 4.2 Baseline regression and heterogeneity analysis

**Figure 3:**
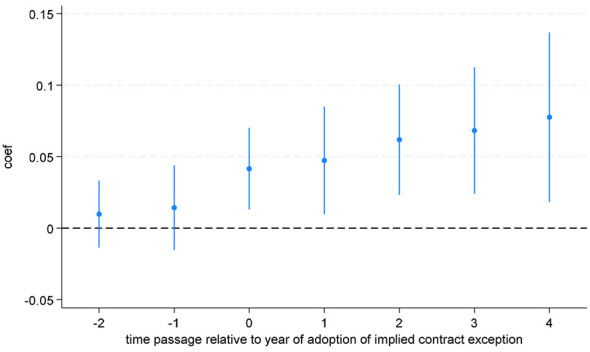
Parallel trend test.

Prior to conducting the staggered DID analysis, this study first performed a parallel trend test to ensure that there were no systematic differences in public health resource allocation efficiency between the treatment and control before the government data platform launch. Using an event study approach with relative time dummies, the results of the parallel trend test are presented in Figure 3. The coefficients for the relative time dummies are not statistically significant before the platform launch, indicating that there were no significant differences between the treatment and control. By contrast, the coefficients for the relative time dummies become positive and statistically significant, with an increasing magnitude over time, suggesting a clear and growing policy effect of government data platform launch. Thus, the parallel trends assumption is satisfied.

Table 3 presents the baseline regression and regional heterogeneity results.

**Table 3:** Regression results.

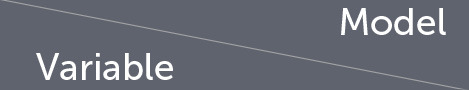	All sample	All sample	Eastern region	Central region	Western region
Public data openness	0.0267^**^ (0.0099)	0.0310^***^ (0.0091)	0.0281 (0.0172)	0.0098 (0.0058)	0.0486^**^ (0.0181)
Per capita GDP	–	−0.0850 (0.0744)	0.0932^*^ (0.0478)	−0.1773^*^ (0.0867)	−0.2556^*^ (0.1137)
Fiscal autonomy	–	0.2688^*^ (0.1496)	0.0472 (0.0806)	0.2237^*^ (0.1119)	0.7181^*^ (0.3450)
Financial investment in public health	–	0.3929 (0.6142)	0.0612 (0.3215)	0.4059 (0.2707)	1.8413 (2.0542)
Child dependency ratio	–	−0.0078^**^ (0.0035)	−0.0024 (0.0037)	−0.0024 (0.0033)	−0.0127^***^ (0.0030)
Old age dependency ratio	–	0.0015 (0.0027)	−0.0003 (0.0027)	−0.0059^*^ (0.0030)	−0.0020 (0.0088)
Urbanization rate	–	−0.0644 (0.2335)	−0.0053 (0.3976)	−0.8531^**^ (0.2628)	−0.4674 (0.7967)
Digital economy policy support intensity	–	−0.0006^**^ (0.0003)	−0.0007^*^ (0.0004)	−0.0002 (0.0004)	−0.0008 (0.0006)
Intercept term	0.9082^***^ (0.0068)	1.8031^**^ (0.7190)	−0.0457 (0.6730)	3.0748^**^ (0.9168)	3.5898^**^ (1.2096)
Province fixed	Yes	Yes	Yes	Yes	Yes
Year fixed	Yes	Yes	Yes	Yes	Yes
Province-specific time trends	Yes	Yes	Yes	Yes	Yes
Sample size	406	406	154	112	140
R^2^	0.4282	0.5081	0.6045	0.5824	0.5473

(1) The clustering standard error is indicated in parentheses. (2) ^*^^**^ and ^***^ denote statistical significance at the 10%, 5%, and 1% significance levels, respectively.

Based on the all sample results, Column 2 of Table 3 reports the regression results including only the core explanatory variable and fixed effects. The impact of public data openness on public health resource allocation efficiency is significantly positive at the 5% level. After incorporating control variables, Column 3 of Table 3 shows that the effect remains significantly positive at the 1% level, with a coefficient of 0.0310, indicating that public data openness significantly enhances public health resource allocation efficiency at the current stage.

Among the control variables, it is noteworthy that fiscal autonomy is significantly and positively associated with public health resource allocation efficiency at the 10% significance level. A plausible reason is that excessive administrative intervention and cumbersome approval procedures may undermine the flexibility and autonomy of healthcare governance, preventing government departments and medical institutions from responding promptly to market changes and social demands, which is consequently detrimental to the resource allocation efficiency. In addition, the coefficient of digital economy policy support intensity is −0.0006 and statistically significant at the 5% level. The weak negative coefficient may reflect short-term resource crowding-out from digital economy investment. Stronger digital economy policy intensity may divert fiscal and administrative resources toward digital infrastructure and industrial development, temporarily crowding out resources dedicated to health resource allocation.

With regard to regional heterogeneous, the effect of public data openness on public health resource allocation efficiency varies significantly. The estimated coefficient is significantly positive in the western region, whereas the effects are insignificant in the eastern and central regions. This heterogeneous pattern may be attributed to the varying relative scarcity of data resources measured by the launch of government data platforms across regions. The western region has a weak foundation in public health resource allocation, prominent data barriers and substantial room for efficiency improvement. Therefore, public data openness generates a more prominent enhancing effect by reducing information costs and improving the supervision system, reflecting a significant latecomer advantage. By contrast, the eastern region has already attained a high level of public health governance and digitalization, approaching the efficiency improvement boundary, thus presenting a weak marginal effect. The central region exhibits a “sandwich layer” characteristic. Compared to the eastern region, its governance capacity is lower. Compared to the western region, it lacks sufficient policy support or room for efficiency catch-up. Thus these limit the effectiveness of policies.

### 4.3 Robustness tests

To further verify the reliability of the regression results, this study conducts a series of robustness tests, with the results reported in Table 4.

**Table 4:** Robustness tests.

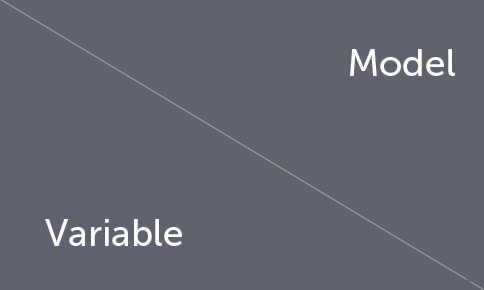	Adjust estimation method			Adjust regression sample	Replace dependent variable	Replace explanatory variable
	CS-DID	PSM-DID	Panel Tobit			
Public data openness	–	0.0365^***^ (0.0107)	0.0310^***^ (0.0076)	0.0222^*^ (0.0129)	0.0336^***^ (0.0121)	–
Public data openness lags by one period	–	–	–	–		0.0188^**^ (0.0070)
ATT	0.0254^**^ (0.0136)	–	–	–	–	–
Pre_avg	0.0156 (0.0121)	–	–	–	–	–
Post_avg	0.0259^**^ (0.0144)	–	–	–	–	–
Control variable	Yes	Yes	Yes	Yes	Yes	Yes
Province fixed	Yes	Yes	Yes	Yes	Yes	Yes
Year fixed	Yes	Yes	Yes	Yes	Yes	Yes
Province-specific time trends	Yes	Yes	Yes	Yes	Yes	Yes
Sample size	368	398	406	319	406	377
R^2^		0.5995		0.5288	0.5221	0.4852

(1) The clustering standard error is indicated in parentheses. (2) ^*^^**^ and ^***^ denote statistical significance at the 10%, 5%, and 1% significance levels, respectively.

First, we adjust the regression method. In this section, we replaced three forms of baseline regression. (1) The baseline regression may suffer from negative weighting bias in staggered DID (36). The CS-DID addresses this issue by estimating the average treatment effect on the treated using only never-treated or not-yet-treated units as valid controls for each treated cohort. As shown in Column 2, the coefficient of the core explanatory variable is 0.0254 (*p* < 0.05). This confirms that our main findings are not driven by negative weighting bias in staggered DID. Besides, we estimate event-study specifications using the CS-DID estimator and report both pre-treatment and post-treatment average effect to explain dynamic treatment effects and validate the parallel trends assumption. It also confirms that our main result is not driven by pre-existing trends. (2) To address pre-treatment systematic differences between treated and control provinces, such as disparities in digital governance capacity, economic development, and health resource endowments, we use PSM-DID to mitigate selection bias. Column 3 reports the coefficient is 0.0365 (*p* < 0.01). The coefficient is slightly larger than the baseline estimate and confirms that our findings are robust to selection bias and pre-existing provincial heterogeneity. (3) Given that the dependent variable (public health resource allocation efficiency) ranges between 0 and 1, we re-estimate the baseline regression model using the panel Tobit model. The results show that the coefficient of government data openness remains significantly positive at the 1% significance level.

Second, we adjust the regression sample. Considering the enormous shock of the COVID-19 pandemic on economic and social development, we exclude the sample after 2020. The coefficient of government data openness is still significantly positive at the 10% significance level.

Third, we replace the dependent variable. The baseline regression adopts the comprehensive technical efficiency estimated by the DEA-CCR model. For robustness, we use the pure technical efficiency estimated by the DEA-BCC model to rule out the interference of scale effects. The regression results indicate that the coefficient remains significantly positive at the 1% significance level.

Furthermore, a key limitation is that our core explanatory variable is a binary indicator of platform launch. It may capture policy initiation rather than the detailed differences in the quality of data openness. While continuous indicators exist, such as the China Open Data Forest Index. These indicators cover only a short period and cannot be applied to our full sample. To address this issue, we further conduct a robustness test by using the 1-year lagged public data openness variable (*L.DATA*_*it*_) to ensure our estimates capture substantive platform operation rather than nominal policy initiation. The results indicate a significant positive effect, consistent with the baseline model. Future research may explore heterogeneity across platforms with different development levels when long-term continuous indicators become available.

**Figure 4:**
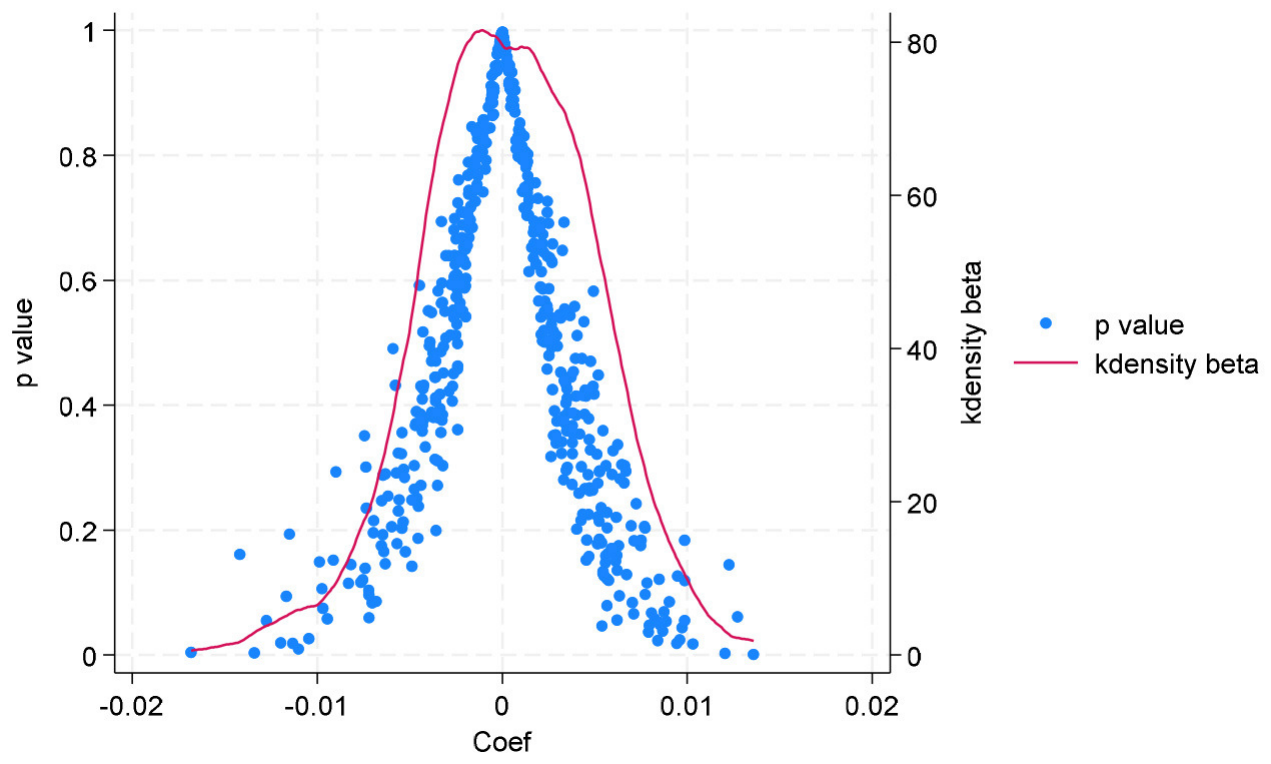
Placebo test.

In addition, to ensure that the findings are not driven by factors other than the launch of open government data platforms, this study further conducts a placebo test. Specifically, we randomly select a pseudo-treatment group with the same number of observations as the original treatment group, randomly generate the policy implementation time, and construct a pseudo-policy variable to regress on public health resource allocation efficiency. This procedure is repeated 500 times, and the kernel density plot of the estimated coefficients. As shown in Figure 4, the estimated coefficients are concentrated around zero, far from the baseline coefficient of 0.0310, and most *p*-values above the 0.1 threshold. Thus, the placebo test was passed.

### 4.4 Exploratory channel analysis

Table 5 presents the results of the exploratory channel analysis. Public data openness has a significantly positive association with the innovation environment utility value and the fiscal transparency index at 5% significance levels. These findings provide suggestive evidence that public data openness may enhance public health resource allocation efficiency through two plausible pathways: reducing information acquisition costs and strengthening external oversight.

**Table 5:** Exploratory channel analysis.

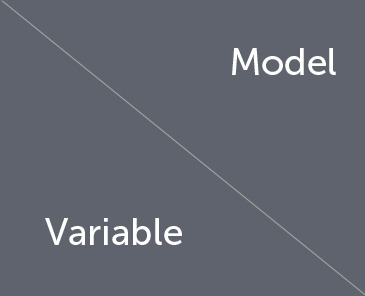	Dependent variable: innovation environment utility value	Dependent variable: fiscal transparency
Public data openness	0.8982^**^ (0.4406)	1.2642^**^ (0.6231)
Control variable	Yes	Yes
Province fixed	Yes	Yes
Year fixed	Yes	Yes
Province-specific time trends	Yes	Yes
Sample size	406	406
R^2^	0.7814	0.7651

(1) The clustering standard error is indicated in parentheses. (2) ^*^^**^ and ^***^ denote statistical significance at the 10%, 5%, and 1% significance levels, respectively.

## 5 Conclusions and implications

### 5.1 Conclusions

This study treats the launch of Chinese government data platforms as a quasi-natural experiment, employs provincial-level panel data from 2009 to 2022, applies DEA, staggered DID, and other methods to examine the effects and transmission pathways of public data openness on public health resource allocation efficiency. The main findings can be summarized as follows. First, public health resource allocation efficiency in China has generally increased in recent years, with inter-provincial disparities gradually converging. Second, public data openness significantly enhances public health resource allocation efficiency. Third, the effect of public data openness on public health resource allocation efficiency exhibits notable regional heterogeneity, with a more prominent efficiency-improving effect in underdeveloped regions. Fourth, exploratory channel analysis suggests that public data openness is plausibly associated with enhanced public health resource allocation efficiency by reducing information acquisition costs and strengthening the external oversight system.

### 5.2 Policy implications

Based on the research findings, the following policy implications are proposed to fully realize the enabling value of public data openness and enhance public health resource allocation efficiency.

First, establish a standardized data governance system and strengthen the synergy between the breadth and depth of data openness and privacy protection. The enabling value of public data openness depends on a governance system characterized by broad coverage, in-depth utilization, and security. In terms of coverage, essential data such as healthcare resource and health outcome, as well as management data including detailed fiscal health expenditures and resource utilization efficiency, should be included in the mandatory open data catalog to ensure full coverage of input and output processes. In terms of depth, the model of shallow data download should be broken through, and desensitized micro-level data should be opened appropriately. In terms of supervision, dynamic monitoring platforms should be established, public supervision channels should be widened, third-party evaluations should be introduced, and the assessment results should be incorporated into the performance appraisal of local governments, so as to force the efficient and compliant allocation of resources.

Second, implement regionally differentiated empowerment strategies to promote fair distribution of policy dividends across all areas. The policy effects of public data openness are influenced by factors such as regional digital infrastructure and governance capacity, resulting in significant variation across different development stages. For developing regions and low-income economies, it is essential to strengthen resource integration and leverage latecomer advantages. Priority should be given to supporting the digital transformation of primary healthcare institutions, establishing regional unified data-sharing platforms, and enhancing local data governance and application capacity through technology transfer and workforce training. For high-income economies with abundant resource endowments, the focus should be on efficiency optimization and model innovation, encouraging the exploration of data-driven smart service models to address resource redundancy and efficiency. For middle-income economies in transition, the focus should be on institutional coordination and resource integration. While drawing on the digital governance experiences of high-income economies, these economies should also take into account their own development realities and narrow the gap in resource allocation.

## Data Availability

Publicly available datasets were analyzed in this study. This data can be found here: https://data.cnki.net.
